# Review on Cold-Formed Steel Connections

**DOI:** 10.1155/2014/951216

**Published:** 2014-02-03

**Authors:** Yeong Huei Lee, Cher Siang Tan, Shahrin Mohammad, Mahmood Md Tahir, Poi Ngian Shek

**Affiliations:** ^1^Faculty of Civil Engineering, Universiti Teknologi Malaysia, 81310 Johor Bahru, Johor, Malaysia; ^2^UTM Construction Research Centre (UTM-CRC), Universiti Teknologi Malaysia, 81310 Johor Bahru, Johor, Malaysia

## Abstract

The concept of cold-formed light steel framing construction has been widespread after understanding its structural characteristics with massive research works over the years. Connection serves as one of the important elements for light steel framing in order to achieve its structural stability. Compared to hot-rolled steel sections, cold-formed steel connections perform dissimilarity due to the thin-walled behaviour. This paper aims to review current researches on cold-formed steel connections, particularly for screw connections, storage rack connections, welded connections, and bolted connections. The performance of these connections in the design of cold-formed steel structures is discussed.

## 1. Introduction 

Light steel framing is referred to as steel frame building constructed with galvanised cold-formed steel sections. As one of the industrialized building systems (IBS), light steel framing has become a popular construction choice in low to medium rise building and residential house construction because it provides numerous advantages as compared to traditional construction methods. To highlight a few: rapid and dry construction, high quality controlled, time and cost saving, accelerating sustainable development by reducing the dependance on timber materials, and minimizing construction wastes.

Structural steel is generally divided into hot-rolled sections and cold-formed sections. These two types of steel sections are distinguished by the method of forming in their manufacturing process. Hot-rolled steel sections are formed under high temperature up to 1400°C in blast furnace or electric arc furnace, while the cold-formed steel sections are manufactured in room temperature. The difference of the manufacturing process makes the properties be of hot-rolled and cold-formed steel disparity in strength, structural performance, and failure mode. Typical thickness of cold-formed steel section is ranged from 0.9 mm to 3.2 mm. The steel is usually galvanized to protect the members from corrosion which will make it more durable. The strength of cold-formed steel is higher than hot-rolled steel per unit weight. In the forming process, due to cold working by the process of strain hardening, the yield strength of the steel will increase [1]. There are three methods to manufacture the cold-formed steel sections: cold roll forming, press braking, and bending brake operation. Among them, cold roll forming is widely used to produce building components (structural members, roof truss, wall panel, frames of windows and doors, etc.). The speed rate of rolling is slow in the range of 6 m/min to 92 m/min [1] to minimised residual stress formation in the notch and edge of the steel section, which would affect the strength of the cold-formed steel sections.

In the past, cold-formed steel sections were used as secondary structural members, for example, roof purlin and side rail for wall cladding. Thin-walled behaviour limits the structural performance of cold-formed steel sections by premature buckling and instability. Over the past two decades, the increasing application of cold-formed steel in construction industry has brought massive researches in progress to ensure the stability and reliability of the constructed steel structures. As the industry demand grows gradually, many research studies were done to minimize the safety issue and exhilarated the use of cold-formed steel members as primary structural members, for example, roof truss, beam, and column member. The research includes the investigation of buckling properties of cold-formed steel members, failure mode of the members, and strength and stiffness of the cold-formed steel member. These research studies provide the latest models and contribute important information to the design guidelines of British Standard 5950 Part 5 and Eurocode BS EN 1993 Part 1.3 and Part 1.8 [2–4]. The situation is obvious in the United States of America which accelerated by the development of research institutes such as American Iron and Steel Institute (AISI) [1, 5].

Connection is defined as the physical component which mechanically fastens the structural elements and concentrated at the location where the fastening action occurs [3]. It is important in transferring force and moment from a structural member to the supporting elements. Joint is defined as the connection plus the corresponding zone of interaction between the connected members and the panel zone of the column web. Structural joints can be classified into several categories by referring to its strength and stiffness. According to Eurocode 1993 Part 1.8 Clause 5.2.3 [3], the strength of joints can be divided into nominally pinned, partial strength, and full strength classification. For stiffness behaviour, nominally pinned, semi-rigid, and rigid connections are classified accordingly to EC 3-1-8 Clause 5.2.2 [3].

The connections for hot-rolled steel structures are divided into bolted connections and welded connections. From these two basic components, numerous joint profiles are developed. Systematic design procedures for these joint profiles are provided for practising engineers [6–12]. Whilst for cold-formed steel structures, there are nine types of joint that are commonly used in the construction industry [1], namely, bolts, self-tapping screws, blind rivets, powder actuated pins, spot welding, puddle welding, clinching, self-piercing rivets, and nailing. The different types of connection contribute to different applications; for example, self-tapping screws, blind rivets, and powder actuated pins are used to fasten thin sheeting to sections, bolts are used to connect thicker cold-formed section, spot welding is used as factory joining of thin steel, and so forth. The characteristics of steel joint are easy to install, ready in the market, of low cost of installation, and with less maintenance needed.

Unlike joints for hot-rolled steel structures, design guidelines for cold-formed steel connections are limited to their fundamental behaviour [1–5]. Detailed design procedures for cold-formed steel joint profiles are uncommon due to their wide variance and specific purpose, and most designs are made based on testing results. Since many researches have been carried out and as the significance of cold-formed steel is concerned, this paper aims to collect and review on researches of cold-formed steel connection from the past two decades. The review is focused on screwed connections, storage rack connections, welded connections, and bolted connections for cold-formed steel sections. The performance of these connections application is discussed.

## 2. Researches on Cold-Formed Steel Connections

Review on the development of cold-formed steel structures has been made [13, 14] in the beginning of the current millennium. The reviews focused on cold-formed steel section behaviour, for example, compression member, distortional and element buckling, corrugated and curved panels, flexural members and purlins, torsion and distortion, web crippling, mechanical properties, composite and plasterboard construction, storage racks, and design optimisation [13, 14]. Minor discussion was made on the connections and fasteners [13]. New design rules for 1.0 mm thickness of mild steel and G550 steel cold-formed steel bolted connection were proposed, with changes made on the bearing coefficient. Tearing of sheet steel combined with bearing failure of bolted connection as well as screwed connection may occur simultaneously. New type of joint, namely, Rosette joint, was introduced in 2000, particularly applied for cold-formed trusses. Rondal [13] discussed on the stability of cold-formed steel members and joints between cold-formed members. In the joints discussion, it was found that press joint and Rosette joint were introduced to the industry in the late 20th century and early 21st century, respectively. Finite element models are used to represent the actual case since the testing in full scale of connection can be very costly. In a connection session discussion, Jaspart [15] suggested that researchers should follow various shape profiles instead of concentrating the investigation on I-beam profile. These new concepts must be applied to industry practically.

From [13–15], the review process was done mostly for cold-formed structural elements. More reviews are therefore made for cold-formed steel connections in the following discussion. The connections are classified as screw connections, storage rack connections, welded connections, and bolted connections.

### 2.1. Screw Connections

Screw is a common type of connections that is used in cold-formed steel. Due to the thinness of the cold-formed steel, screw connection provides advantages in simple design and fast installation. Screwed joints are suitable and effective when applying into the cold-formed steel section with the condition that total thickness should not give difficulty to the self-drilling process.

The development and testing of self-drilling screw were discussed [16]. Experimental tests were conducted for cold-formed sections with drilling screws subjected to single shear. Self-drilling screw showed better moment of capacity and rigidity as compared to conventional joints [17–19], when bolted end plate moment connection was found impractical to apply in portal frames knee joints. The research showed that the proposed self-drilling screw joints could overcome the effective modulus properties (different lips, flanges, and webs dimensions) for both Australian and American sections. Screwed ridge joint was also studied under wind at roof pitch condition [17]. Gusset plate was replaced with the channel section and connected with different configuration of self-drilling screws in order to obtain the structural behaviour of screw joints.

Design equations were first developed by Peköz [20] on screwed connection. The developed equations were based on an analysis of thousands of connection test data. The strength of screw connections in cold-formed steel construction was investigated [21] together with the safety and resistance factors. The strength of screw joints was lower than the connected members. Designers were advised to use the safety factor provided by manufacturer when using their material. Tilt fastener was recommended to be eliminated during screw shear strength tests for more accurate shear strength of a fastener.

Moreover, the seismic design and fire resistance design for screwed joints were discussed [22–25]. The X-braced shear walls with NSF (net section failure) connection were needed to design in seismic zone. The study on screwed joint in straps with experimental data was presented. Elastic stiffness and tangent stiffness of the joint and the strap must be considered in design. Cold-formed roof sheeting connection with self-tapping screw was investigated at ambient and elevated temperatures [23]. The effect of edge and end distance on the connection resistance was studied by using finite element method and experimental investigation. At ambient temperature, 2.5 of reduction factor could be used for shear-out failure. The equations have been revised for the ratio of the edge distance to the diameter that is in the range of 1.00–1.75. Partial safety factor was derived when taking temperature effects into account. In Hong Kong, there was a research program that studied the screwed connections of thin sheet steel at elevated temperatures using steady state test and transient state test with a total of 102 specimens [24, 25]. The observed failures included bearing, tilting and bearing, screw shear, net section tension, tear out failure and material failure. It was found that current design specifications are conservative towards strength prediction at elevated temperatures. For transient state test, failure mode of the screwed connection may change as the load increases.

There was a series of full-scaled isolated screwed joint tests investigated in Universiti Teknologi Malaysia [26]. A total of 12 beam-to-column connections were studied with different configurations and compared with Eurocode analytical model. Initial stiffness of the joints increased as the beam depth increased. The investigated screwed joints were all considered as partial strength category when classified according to their strength, as the strength of joint was more than 25% of connected beam flexural resistance.

### 2.2. Storage Racks Connections

**Figure 1:**
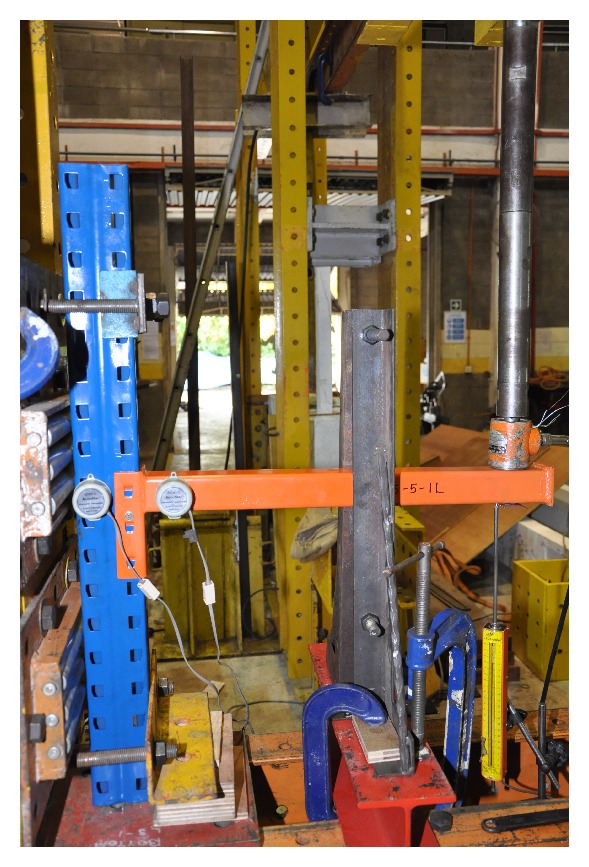
Typical flexural test for storage rack connection.

There are many types of rack structures such as pallet racks, drive-in racks, drive-through racks, cantilever racks, selective pallet racks, narrow aisle racks, double deep pallet racks, push-back racks, and gravity flow and pallet flow racks. Storage rack structures are mainly designed by cold-formed steel members. Since the rack systems are similar to light steel frames, the parameters involved in design are also mostly equivalent.  Figure 1 shows an example of the test setting up for flexural behaviour of the connection for storage rack. The influence of beam-to-column joint on the frame response is investigated under service limit state and ultimate limit state [27]. Semicontinuous frame, as an optimum model, was studied for the actual behaviour of beam-end connectors which contributes to the lateral stiffness of the frame.

The behaviour of the key components of racks was evaluated and the main parameters in its performance were assessed in terms of beam-to-column joints [28]. The design guidelines for the seismic zones of the racks were highlighted in cyclic experimental tests with strength degradation and energy dissipation capabilities which can influence the connection in the form of the hysteresis loops. Simplified model will be developed for racks design in future. Correction equations were derived for sway behaviour under a combination of point loads with increasing side loads [29]. For FEM (Federation Europeene de la Manutention) code, the predicted lateral deflections were overestimated but accurately predict joint resistance, whereas, for SEMA (Storage Equipment Manufacturers' Association) code, the lateral deflections were underestimated and unable to predict joint resistance.

Instead of single cantilever, double cantilever tests were conducted to determine the flexibility of beam-column connectors in conventional pallet racking systems in hook-in end connectors by finite element method [30]. Double cantilever has better illustration in actual frame shear to moment ratio. Steel pallet rack systems or structures have a great experience subjected to seismic load [31]. From semirigid connection experimental study (conventional cantilever method and double cantilever method) and finite element model analysis, an analytical model was developed to describe the seismic behaviour in down aside direction. Pallet racks are considered as slender structures which are sensitive to the second order *P*-Δ effect.

At certain occasion, bolted moment connections are economical and feasible between cold-formed steel members in storage rack system. A study on bolted moment connections in drive-in and drive-through steel storage racks [32] was carried out. From the research, finite element results showed that the moment of bolted portal beam to upright connection at the design load is lower than the inducing slippage in the connection. Hence, the looseness in the connection could be neglected. Furthermore, the flexibility of the steel racks (boltless and semirigid in nature) was investigated by using finite element method [33]. A general Frye-Morris type (three-parameter-power model type) moment versus relative rotation relationship was developed. The most influencing parameters are thickness of column, connector depth, and beam depth. The initial connection stiffness and shape parameter were obtained by using the ultimate moment capacity. The three-parameter-power model was proposed to represent the moment-rotation behaviour of the boltless connections.

Connections and base plates were the most significant factors that affect the structural behaviour of drive-in racking system [34]. An equation was developed and compared with experimental data and numerical results. The limit moment from the proposed equation can be employed in design procedures. Elastic stability analysis of cold-formed pallet rack system was investigated with semirigid connections [35]. Experimental results, calculation from effective length approach, and finite element results were compared. From the study, it was found that stiffening of the open upright sections could enhance the load-carrying characteristic of the frames by using the spacer bar, channel, and hat as additional stiffeners.

### 2.3. Welded Connections

Welded joints provide rigid joints between the cold-formed steel members. Welding operation involves skilled workers. Welding served with extra care as compared to other joints is discussed. The review focuses on laser beam welding (LBW) [36–41], stainless steel welding [42, 43], and other welded joints [44–51].

Laser beam welding (LBW) is widely used in automotive industry, yet it is still considered as new term in cold-formed steel construction. A study of possibility of introducing the LBW technology into cold-formed steel connection was initialled and continued with a massive laboratory testing [36–40]. In the study, experiment tests were consisting of element tests (lap-shear and *U*-tension tests) and beam flexural tests. The investigation was aimed at examining the innovative connection in building up cold-formed steel beams. The LBW technique is precisely welded to the joints which will minimise the error and thus increase the safety of the connections. For connection configuration on the flanges, welds with spacing of 100 mm have the same flexural resistance with spacing of 50 mm. The failure characterization of laser welds was investigated under combined loading conditions [41]. The failures were divided into two regions, namely, base metal failure and interfacial failure.

A study was made on tubular X-joint with the carbon steel replaced by stainless steel [42]. Since stainless steel does not have a distinct yield stress, 0.1%, 0.2%, 0.5%, and 1.0% of proof stresses were included in the investigation. Three failure modes occurred as follows: chord face failure, chord side wall failure, and local buckling failure of brace. The investigation showed that the 0.2% of proof stress is reasonable in predicting the design strength of stainless steel tubular X-joint in rectangular hollow section for both ultimate limit state and serviceability limit state. The prediction may replace the lack of design guideline in Australian/New Zealand Standard. A research on stainless steel tubular T-joint of square and rectangular hollow section brace and chord members was conducted [43]. The paper agreed to the recommendation by [42] that the 0.2% of proof stress is reasonable in predicting the design strength of stainless steel tubular T-joint in rectangular hollow section, for both ultimate limit state and serviceability limit state.

Moreover, T-joints in square or rectangular hollow sections (SHS or RHS) and circular hollow sections (CHS) have been investigated. A web buckling formula was proposed to predict the deformation limit and ultimate strength in RHS [44–48]. In predicting the web buckling strength and chord flange strength, the corner radius of RHS was considered. There were studies that investigated the fatigue tests and design in tube-to-tube joints [49] and section-to-plate connections [50] of SHS under in-plane bending. In tube-to-tube joints, three options have been proposed in nodal concentration design (where failure occurs in tubes of thickness equal to 3 mm). The fatigue life of SHS section-to-plate connections decreases as the thickness of the section decreases from 4 mm and curve was proposed with 2.0 recommended SCF (stress concentration factors) for the hot spot stress [51]. The section-to-plate T-joint of CHS was investigated under in-plane bending [52]. CHS T-joint has better fatigue strength as compared to SHS T-joint. Arc welded T-joint was investigated with a series of laboratory tests [53]. An equation was proposed to represent the strength of the weld. The behaviour of the SHS T-joint under in-plane bending was studied using finite element analysis [54]. A model was developed to study the sensitivity of parameters. The plastic behaviour of knee joint of RHS was investigated [55]. From the research, it was found that the internal sleeve connection was suitable in plastic design.

### 2.4. Bolted Connections

Bolted connection is a common fastener in steel construction which can be applied to both hot-rolled and cold-formed steel. For bolted connection, the research can be divided into several sessions such as stainless steel, lapped Z-purlins, and seismic design.

Winter proposed design equations for cold-formed carbon steel bolted connections [56]. The net section and bearing resistance were applicable to light steel framing design. Experimental investigation for bolted connections between thin gauge stainless steel was carried out [57]. The carbon steel design provisions are suitable in predicting tear-out failure and net section rupture but overestimate the bearing resistance of bolted connection. Net section failure [58] and bearing capacity [59] were investigated in stainless cold-formed steel bolted connections. The edge distance “*e*_2_” and bolt configuration were the main parameters that affect the net section rupture of bolted connections. A proposed design equation for net section capacity of stainless steel connection was validated by statistical and numerical analysis [58]. There are two proposed equations of bearing design for two conditions. The first condition is controlled by fracture, whereas the second condition considers the deformation under service loads [59].

A finite element model consists of bolted connections in thin-walled stainless steel plates under static shear was developed [60]. The Japanese steel design standards (AIJ) have less accuracy in predicting the ultimate behaviour (failure mode, ultimate strength, and the occurrence of curling) of stainless steel bolted connections loaded in static shear. The paper proposed a modified formula for calculating the ultimate strength. The load-displacement curves of bolted connections are predicted with nonlinear material and nongeometric finite element analysis. The finite element analysis was used to analyse the structural behaviour of single shear bolted connections with two bolts in cold-formed steel structures [61]. The ultimate strength, failure mode, and curling of the finite element model give reasonable agreement in comparison to experimental data. It can be found that Kuwamura's equations from Stainless Steel Building Association of Japan (SSBA) are more adequate when estimating the ultimate strength of bolted connections with no curling. The ultimate strength in SSBA design standard of the connection was overestimated in curling effect case. A finite element analysis met good agreement with the experimental data [62]. The effect of curling on bolted connection was considered in revised design formulae that are then proposed in the paper by using correlations between strength reduction ratio and plate thickness.

Nevertheless, the behaviour of bolted connection in multispan of purlin was investigated [63–67]. Rigid connection was assumed in old practice for the ease of calculation. With laboratory tests as evidences, the research was conducted in semirigid in bolted lapped connection. It was found that different failures were formed when semirigid connection was assumed. In [63], the wed crippling action at the edge of the lapped zone was taken into account with bending moment in multispan cold-formed Z-purlins with bolted lapped connection. From the paper [63], the lap edge single section was considered as critical in purlin design and lateral-torsional buckling becomes critical when in the case of laterally unrestrained purlins. Critical section of both cases is the edge of the lapped connection. Moment resistance and flexural rigidity of Z-purlin in multispan structure were investigated later [64] and targeted on the moment capacity and flexural rigidity of lapped connection over the internal support in multispan purlins system. Several conclusions were drawn from the research where bending moment was dominating the load-carrying resistance, the critical section was the edge of lapped connection, moment resistance of the edge section of lapped connection was twice less than the moment resistance at internal support section, the effective flexural rigidity was affected by the length of lapped connections, and the effective flexural rigidity of lapped connection could be estimated by using stress analysis and deflection analysis. There were a number of researches completed by Ho and Chung [65–67] on multispan purlins overlapping connection. The researches were about the Z-purlins lapped connection with experimental testing and modelling to figure out the behaviour of the lapped connection. In the researches, structural behaviour of lapped connections of cold-formed steel Z section was investigated with one-point load experimental tests. After the tests were done, an analysis and design method was developed. Hence, an analytical method was developed to predict deformation characteristics of the lapped connections and the structural behaviour of the generic bolted configuration was investigated. The assumption that the moment resistances and flexural rigidities of lapped Z sections have doubled the strength of connected members is not always valid. A rational design method with moment and shear resistance was introduced. The analytical method is important in determining the effective flexural rigidities over internal supports of lapped connections. With the generic bolted configuration, the design rules for multispan purlin systems with lapped connections were set.

Seismic design for bolt connections should be a concern. In [68], an experimental study was conducted on tensile bolted joints between straps. The investigation included the experimental and numerical research on behaviour of dissipative X-braced shear walls. According to the authors, joints can be divided into two groups which were joint fails due to bearing and tearing phenomena (T + B + TS) (tilting, bearing, curling, and tearing of the sheets) and joint fails due to NSF, net section failure (T + B + NSF). Several conclusions can be drawn as T + B + NSF type of joint is preferred due to ductility; the situation can be improved by introducing washers to increase the strength of connection; the equations described in the BS EN 1993-1-3 are considered accurate but conservative and become inaccurate when applied to joints with 8 mm bolts. There were two groups of recommendations given in seismic design of cold-formed steel joints between straps which included increase of the ductility of joints and improvement of the performance of the joints. A claim describes that screws are better than bolts in connecting straps of a dissipative X-braced frame. Seismic design for earthquake event has been proposed by AISI, American Iron and Steel Institute, which is based on the elastic design.

Papers [69, 70] discussed the inelastic bolted moment connections while the beams and columns remain elastic. The aim is to provide information in developing the capacity design provisions in the proposed AISI seismic standard for CFS-SBMF (cold-formed steel structural systems-special bolted moment frames). Bolt slippage and bearing in the bolted moment connection are the source of energy dissipation. From the tested results, a bolted connection would first slip and hence produce a pseudoyield behaviour. The slip force and slip range are then tabulated to facilitate design. Thus, a flowchart is obtained to demonstrate the proposed capacity design procedure for seismic design for CFS-SBMF. Furthermore, the failure modes of the bolted-sheet-steel connections in shear were identified [71]. The load capacity formulations in AISI cannot represent the failure modes in cold-formed steel. The net section fracture and bearing failure modes should be concerned about in cold-formed steel design.

Bolted moment connections which include column base tests and beam-column subframe tests with different configurations were conducted to predict their strength and stiffness [72, 73]. The following conclusion can be drawn in Table 1. Further experimental study from the above investigation was conducted [74, 75]. The analysis and design methodology for internal forces distribution of connections were presented and design rules for both shear and bending of the joints were proposed. With finite element models that have good calibration against test data, a semiempirical formula for flexibility prediction of bolted moment connections was then proposed.

A series of laboratory tests and finite element analysis were carried out to determine the ultimate strength of bolted moment connections between cold-formed steel members [76]. Web buckling was concerned in design to predict the ultimate strength of the connections and cannot be ignored. A method that determines the initial rotational stiffness of shear-bolted moment connections between cold-formed steel members has been presented [77]. The predicted stiffness can be used in frame design with reasonable safety considerations. From the predicted strength and stiffness, beam idealization method was used to predict the structural behaviour of 2D portal frame [78]. A closed comparison with experimental results was found and was able to reduce the computational time while using beam idealization. Besides, a series of connection experimental tests were conducted to investigate the connection performances of a pitched roof portal frame [79]. Flexural strength and structural behaviour (moment-rotation relation, the yield, and ultimate moment capacity) were studied experimentally. Both experimental data and numerical data were conducted. Using secant stiffness, semirigid connection was applied to the analysis which was estimated from moment-rotation curves from tests.

Research was conducted to predict the tensile strength of bolted cold-formed channel section [80]. The overdesign was found in cold-formed steel design with design standards. An equation was proposed to overcome the overestimated design which grabs good agreement with test results. The strength requirements [81], plastic and lateral-torsional buckling behaviour [82] of single cold-formed channels bolted back to back, were investigated. Increase in bolts number did not have significant improvement in moment capacity of the joints. Semirigid bolted connection was considered in plastic design of the back-to-back channels. The research above was more focused on the behaviour of the sections that were built up back to back. Anwer et al. [83–85] have performed a study on bolted moment connection of base connection and beam-to-column connection for single channel section. Several configurations have been studied and were able to achieve partial strength connection behaviour. The study was furthered to frame analysis. The proposed connections are suitable to be applied in the frame design.

A study on the stiffness of joints in bolted connected cold-formed steel trusses was investigated [86–89]. A theoretical model for joint stiffness was proposed with summarized experimental data for several years which can represent the real behaviour of practical trusses. The study on efficiency reduction due to shear lag on bolted cold-formed steel angles was carried out [90]. A two-parameter function (length of the connection and distance of the shear plane to the centroid of the cross section) can be represented to the reduction coefficient. A new expression for the netsection reduction coefficient was suggested. A research was done on experimental investigation of the bearing capacity of cold-formed steel bolted connections using oversized holes without washers [91]. An extended deformation and reduction in strength were found when the situation of oversized holes without washers was occurring. A new design method was developed to predict the behaviour of the connections with situation of oversized holes without washers. A study on cyclic testing and modelling of cold-formed steel special bolted moment frame connections was conducted [70]. A model that can simulate the cyclic behaviour of bolted moment connection was presented based on the concept of the instantaneous centre of rotation of an eccentrically loaded bolt group.

**Figure 2:**
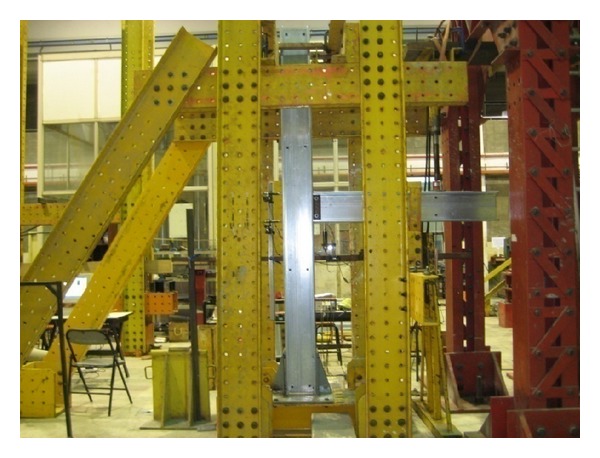
Isolated joint test setup for beam-to-column connection [92].

Tan studied the behaviour of beam-to-column connections [92–95]. Experimental investigation has been performed on double web cleat connection, top-seat flange cleat connection, flange-web-cleat connection, gusset plate connection, and combined gusset plate and flange cleat connection [92].  Figure 2 shows the isolated joint test setup in this investigation. Comparison between Eurocode model, finite element results, and experimental data for top-seat flange cleat connection has been done in order to know the difference using Eurocode [4] as design specification. It was found that there is an inadequacy when adopting Eurocode in the connection with cold-formed channel sections.

## 3. Discussion 

Current research of cold-formed screwed connection is focused on cold-formed steel sheeting due to drilling difficulty for primary structural members. Various screw configurations were studied to obtain optimum resistance strength. Economical design can be achieved by adopting the results from current research. The research is furthered to structural behaviour at elevated temperature.

Screw connection is a convenient method for joining thin steel section or steel sheeting. It does not require heavy and precise tools at site and speed up the prefabrication process. The difficulty of self-drilling process will increase with the increment of steel thickness and steel grade. For secondary structural member, screwed connection has shown the effectiveness in structural member. However, for primary member, the enhanced section in steel grade and section thickness for better resistance has induced the problem of drilling. Since screw connection is applied on site, the connected members should not exceed human controllable length and weight. The increment of in situ machineries use while connecting members will affect the accuracy of screw location. Based on practical installation considerations, typical applications, and the fastener reliability, Mujagic and Easterling recommended that screw is not to be applied for thickness of the thinnest connected part that exceeds 3.2 mm (0.125 inch) [96].

For storage rack connection, base plate connection and beam-to-column connection are the two significant factors that affect the structural behaviour of rack system. Beam-to-column connection is the major direction for current research trend. Both second order effects of *P*-Δ and *P*-*δ* should be taken into the design consideration as the racks are slender structures. Semirigid behaviour of the connection for storage rack system should be included to obtain optimum design. Another common problem that faced rack system is vibration load when loading and unloading materials on the racks. The seismic zone experiences these loading vibrations and the connection must have the fatigue tests to prove the adequacy of design guidelines.

As modular construction becomes more popular in construction sectors, structural beam-to-column connection for building should take the rack system connection as an example for the ease of construction. This type of configuration can accelerate the modular system for fast construction development.

The new technique of laser beam welding (LBW) is introduced to building industry. A massive research should be conducted to find out the strength of this joint on various sections and various connections, the reliability of LBW applied in structures, the influence of LBW types in strength in joints, the influence of heat affected zone, failure mode that may occur, and so forth. Future research on LBW also should be carried out not only on build-up section joints but also on beam-to-beam joints, beam-to-column joints, column-to-column joints, and column-to-base joints.

The heat from welding process will reduce the strength of the cold-formed sections. For cold-formed section welding, the minimum section thickness is roughly in the range of 1.3 mm to 1.7 mm, according to AISI [97]. The impact properties for the welded structure should be investigated with referring to code specifications in order to understand the influence of heat to structural behaviour by experimental study. The thickness of thinner section should not exceed 4.8 mm (0.1875 inch) [5].

For bolted connection, the focus of current research is majorly on the secondary members such as purlin and truss design. Limited references are found for primary loads carrying members, beam-to-column connection, beam-to-beam connection, and column base connection. In the authors' point of view, for ease of construction, the bolted connection is preferable in the construction sector. The reduction of skilled worker at construction site with bolted connection application is able to minimize the construction cost as compared to welded connection.

Bolted connection in cold-formed steel structure is able to achieve semirigid behaviour according to stiffness classification. However, it was unable to reach full strength behaviour as classification of strength to the Eurocode [4] due to the thin-walled structures tends to buckle in compression condition. Semirigid and partial strength behaviours are the suggested area in the trend of cold-formed connection research. According to Eurocode [4], there are some inadequacies in the design as the failure of yield line pattern may not occur in reality. Buckling will occur before the formation of the yield line in cold-formed steel bolted connection.

In most of the current codes of practice, connection design is based on single fastener capacity regardless of the resistance of the connected members. Due to thin-walled behaviour, cold-formed steel exhibits different failure mode and large deformation as the buckling is the major concern of the connection structural analysis. The contributions of each component from the developed joints should be identified to achieve more reliable structural behaviour. This can be done by component method which is currently codified in the Eurocode for three types of connection typologies. However, the formulations in Eurocode, initially developed for hot-rolled steel joints, might be inaccurate when applied to the design of bolted cold-formed steel connections [93–95]. Thus, comprehensive studies should be carried out to improve the reliability of joint design according to codes of practice for light steel framing.

## 4. Conclusion

This paper summarises previous research on cold-formed steel connections in the area of screw connection, storage rack connection, welded connection, and bolted connection. Screw is a common type of connections that is used in cold-formed steel due to the thinness of the cold-formed steel sections. Storage rack is considered as part of light steel framing because both the material and structural configuration are similar to steel framed building. Improved welding technologies, for example, laser beam weld (LBW) and stainless steel weld, are among the active discussion topics on welds for cold-formed steel connection. Bolted connection is a common design; however, extra considerations should be taken for when designing bolted cold-formed steel connections in accordance with Eurocodes. There are some gaps of knowledge that are needed to be filled up on the design codes for the cold-formed steel connections. This could be completed with comprehensive future research.

This review is initiated to summarise researches on cold-formed steel connections. The work is limited by the scopes of discussion as well as the length of the publication. There are several other researches that are worth to be further discussed. For example, continual efforts that are done by the Network for Earthquake Engineering Simulation (NEES) and Federal Emergency Management Agency (FEMA) of the United States would provide more in-depth knowledge on the behaviour of steel connections under seismic behaviour. Some standardized joint configuration proposed by FEMA, even though initially not meant for cold-formed steel structures, the design philosophy might help to fill up the gaps of knowledge. Innovative design of joints for storage racking system and improvement in welding technology for thin-walled cold-formed steel structures are also worth to be reviewed and compiled for knowledge advancement.

## Figures and Tables

**Table 1:** Different failure modes to predict the joint strength and stiffness.

Failure mode	Moment capacities of connected section
BFcsw Bearing failure in section web around bolt hole	<50%
LTBgp Lateral torsional buckling of gusset plate	About 60%
FFgp Flexural failure of gusset plate	About 75%
FFcs Flexural failure of connected steel section	Minimum 85%
